# AM1241-Loaded Poly(ethylene glycol)–Dithiothreitol Hydrogel Repairs Cranial Bone Defects by Promoting Vascular Endothelial Growth Factor and COL-1 Expression

**DOI:** 10.3389/fcell.2022.888598

**Published:** 2022-05-18

**Authors:** Yilong Ai, Wenting She, Siyuan Wu, Qing Shao, Ziran Jiang, Pengcheng Chen, Li Mei, Chen Zou, Youjian Peng, Yan He

**Affiliations:** ^1^ Foshan Stomatological Hospital, School of Medicine, Foshan University, Foshan, China; ^2^ Center of Regenerative Medicine, Department of Stomatology, Renmin Hospital of Wuhan University, Wuhan, China; ^3^ School of Dentistry, University of Queensland, Herston, QLD, Australia; ^4^ Department of Orthodontics, Faculty of Dentistry, University of Otago, Dunedin, New Zealand; ^5^ Institute for Regenerative and Translational Research, Tianyou Hospital of Wuhan University of Science and Technology, Wuhan, China

**Keywords:** bone regeneration, cranial bone defects, PEG-DTT hydrogel, AM1241, osteoblasts

## Abstract

**Objective:** To explore the repair effect of the prepared drug-loaded AM1241 poly(ethylene glycol)–dithiothreitol (PEG-DTT) hydrogel on cranial bone defects in SD rats.

**Methods:** The PEG-DTT hydrogel under borax catalysis was quickly prepared, and the characterization of the material was observed by a scanning electron microscope. The effect of AM1241 on cell activity and bone tissue differentiation was tested. The SD rat model of cranial bone defect was established, and the defect was repaired by injecting the prepared hydrogel into the defect. The defect was divided into four groups, namely, sham group, blank group, PEG-DTT group, and PEG-DTT + AM1241 group. The rats were euthanized, and whole cranial bone was taken out for micro-CT and histological observation.

**Results:** The prepared hydrogel is porous; it is liquid when heated to 80°C and a hydrogel when cooled to 25°C. 5–10 μM AM1241 increased osteoblast activity. A moderate amount of AM1241 can promote osteogenic differentiation. Both the PEG-DTT group and PEG-DTT + AM1241 group showed obvious new bone tissue formation, but the PEG-DTT + AM1241 group had a better effect. In addition, the new bone tissue in the PEG-DTT + AM1241 group was significantly more than that in the other groups.

**Conclusion:** The prepared AM1241-loaded PEG-DTT hydrogel showed a good repair effect on SD rats with cranial bone defects. It can be used as materials for cranial bone repair in SD rats with cranial bone defects, but the repair effect is weaker than that of normal bone. These results provide a theoretical and practical basis for its further clinical application.

## Introduction

In our whole life, the bones support the structure of our bodies and are one of the organs we rely on to carry out our daily activities, whereas bone defect is a general, frequently occurring disease clinically. There are various factors which can lead to bone defects, such as congenital developmental disorders, tumor, trauma, infection, fracture, osteoporosis, and so on (Liang et al., 2020). In general, the bones have the remarkable capacity to repair and heal themselves after trauma and illness, but when the defects are large enough that exceed their ability to repair itself, it will never completely be reinstated (Forrestal et al., 2017). Traditional treatments of bone defects like bone grafts and metal prostheses have their own shortcomings, such as infection, additional surgery, and the necessity for suffering of pain. Meanwhile, bone remodeling is the main process to maintain the integrity of bone structure (Hojnik et al., 2015). Whether it is due to congenital factors or physiological aging or bone defects caused by trauma, we should focus on finding more useful methods or materials to alleviate the inconvenience caused by bone defects.

With the development of science and intensive study of material, bone tissue engineering becomes a promising substitute to traditional methods, namely, autografts, allografts, and xenografts (Lavanya et al., 2020). As a new biomaterial with controllable mechanical properties and biocompatibility, a hydrogel is widely used in bone tissue engineering (BTE) as a scaffold for growth factor transport and cell adhesion. Compared with other biomaterials, hydrogels have similar porous structures to the extracellular matrix and have good biocompatibility. Thus, they can be used as carrier materials for cells or bone growth to promote growth factors in BTE (Buwalda et al., 2017). Moreover, hydrogels provide a three-dimension hydrate environment, which allows the embedding of bioactive factors and even cells. It also supports the colonization of host cells and the entry of new tissues. These materials can effectively fill up the nonuniform shapes of the cavities without causing tissue damage, thus alleviating the suffering of patients and reducing the medical costs associated with surgery (Fenbo et al., 2020). Polyethylene glycol (PEG) is a high molecular polymer with excellent lubricity, moisture retention, dispersibility, and adhesion. The PEG hydrogel has been widely studied in the treatment of bone defects due to its adjustable properties in chemistry and mechanism, as well as many other useful properties (Lu et al., 2018). Dithiothreitol (DTT) is a small-molecule organic reducing agent that acts as a reducing and deprotecting agent for thiolated DNA. In particular, when DTT is added to PEG hydrogels, the hydrogels showed better bioadjustability and could be more applicable to tissue repairs. Moreover, various small-molecule drugs have been used with hydrogels for improving the efficiency of treatment (Hotta et al., 2016). AM1241, the cannabinoid receptors 2 (CB-2) agonist, can promote osteoclast differentiation (Li et al., 2020). Numerous studies have pointed out that the combination of hydrogels and the agonists showed a stronger stimulant effect on bone regeneration (Kim et al., 2014). Meanwhile, as one of the influenced factors of bone remodeling, the endocannabinoid system plays a crucial part in regulating cell proliferation, differentiation, and survival (Galve-Roperh et al., 2013).

Therefore, we designed a novel hydrogel composed of PEG and DTT to work together with AM1241 and validated its effect in animal models. We demonstrated that the AM1241-loaded PEG-DTT hydrogel could stimulate the osteogenesis based on the calcium mineralization, providing a new method to support for bone regeneration.

## Materials and Methods

### Preparation of Hydrogels

PEG-DTT hydrogels were prepared by the Michael addition reaction using poly(ethylene glycol) diacrylate (PEGDA) (molecular weight: 700, Sigma-Aldrich, United States) and DTT (Sigma-Aldrich, United States) over the sodium tetraborate catalyst. AM1241 (0, 1, 2.5, 5, 10, 20, 40, 80, 120, and 200 µM) (Sigma-Aldrich, United States) was then immediately added to this solution and mixed to prepare a finished AM1241-loaded PEG-DTT hydrogel. First, 100 mg of PEGDA (0.14 mM) and 22 mg of DTT (0.14 mM) were dissolved in 0.5 ml of water. Then, 0.5 ml of 0.1 mol/L borax (Sigma-Aldrich, United States) solution was added to the mixture and stirred vigorously for 10 s. Keep the solution at room temperature (25°C) for a while and check the gelation time by tube inversion.

### Observation of Hydrogel Morphology

The morphology of the PEG-DTT hydrogel was observed by a scanning electron microscope (SEM). The PEG-DTT hydrogel samples were prefrozen in a −20°C freezer and then freeze-dried at −52°C for 72 h using a freeze dryer. The hydrogel samples were cross-sectioned and fixed on metal stubs with carbon tape. Then, the cross-section of the dried sample was sprayed with gold in vacuum. The samples were sputtered coated with gold/palladium for 30 s on a Denton Desk II sputter coater with global rotation and tilt. The surfaces of the gold/palladium-coated hydrogels samples were observed at 5–8 random locations per sample (*n* = 3) on a Zeiss Ultraplus thermal field emission SEM at 10 kV.

### Thermosensitive Properties of Poly(ethylene glycol)–Dithiothreitol Hydrogel

The PEG-DTT hydrogel was dissolved in phosphate buffer solution (PBS), and the solution was made of 4 wt%. Placing 200 μl of the solution in a 2 ml glass bottle at room temperature, heat the bottle and stir the solution to 80°C. Then, cool it naturally to room temperature of 25°C. Every time the temperature changes, the glass bottle was tilted and photographed to record the state of the hydrogel.

### Cell Culture, Toxicity, and Proliferation Assay

Rats bone marrow mesenchymal stem cells (BMSCs) were obtained from the marrow of healthy 4-week-old Sprague-Dawley (SD) rats. Additionally, seeded the cells in T25 culture flasks (ThermoFisher, Shanghai, China) and maintained the flask with a-MEM (HyClone, Shanghai, China) at 37°C and 5% CO_2_, supplemented with 1% penicillin/streptomycin (Gibco, Shanghai, China) and 10% foetal bovine serum (FBS, Gibco, Shanghai, China).

We have used the cell counting kit-8 (CCK-8, Dojindo, Kumamoto, Japan) assay to measure the viability of cells, which were cultured in various concentrations of AM1241. Cells were seeded in 96-well plates at a concentration of 5,000 cells per well and cultured with increasing concentrations of AM1241 (0, 1, 2.5, 5, 10, 20, 40, 80, 120, and 200 µM) for 24 h. Then, 100 µl of a-MEM and 10 µl of CCK-8 were mixed and added to each well after culturing for 6, 12, or 18 h. The absorbance of the wells at a wavelength of 450 nm was measured on a microplate reader (Mode 680, Bio-Rad, Hercules, United States) after 1 h incubation at 37°C.

Osteogenic differentiation medium (Cyagen, Guangzhou, China) was used to induce the procedure of osteogenic differentiation, and all the procedures are carried out according to the user manual. Briefly, cells of BMSCs were seeded in 24-well plates and cultured in the abovementioned osteogenic differentiation medium containing several concentrations of AM1241 (0, 5, and 10 µM). We divided them into four groups, namely, control group, osteogenic differentiation medium group, osteogenic differentiation medium + 5 μM AM1241 group, and osteogenic differentiation medium + 10 μM AM1241 group. Replaced the osteogenic differentiation medium every day and measured the calcium deposits by Alizarin red (AR) staining (Cyagen Biosciences, Guangzhou, China) every 7 days until 3 weeks of culture. The specific steps of AR staining are described as follows: tissue was fixed with ethanol solution for dehydration and embedding, sectioned, and placed in 95% ethanol. After air-drying, AR-containing staining solution was added to the sections and stained by immersion for 5–10 min after which followed by a quick rinse using distilled water. Routine dehydration and transparency were followed by sealing using neutral balsam. The medium was removed from the plates, washed 2 times with PBS, and then fixed with 4% paraformaldehyde (Cyagen Biosciences, Guangzhou, China) for 10–15 min. Remove the fixative and wash 3 times with ddH_2_O. After drying water, AR staining solution was slowly added and stained for 20–30 min. Remove the dye and then wash with ddH_2_O for 3–5 times. Add an appropriate amount of ddH_2_O to each well to prevent the wells from drying, observe under the microscope, and take pictures.

### Animal Model

Twenty male SD rats were purchased from Vital River Laboratory Animal Technology Co. Ltd. (Beijing, China). Animals were housed in a specific pathogen-free grade environment and fed with normal water and food. All procedures conformed to animal experimentation standards and were approved by the Ethics Committee of School of Medicine, Foshan University.

In order to set up the model of rat cranial defect, 20 about 8- to 9-week-old Sprague-Dawley male rats were placed under anesthesia. The surgical area was disinfected with iodophor twice. Then, a longitudinal skin incision was made along the midline of the rat cranial bone (sagittal suture), about 1.5 cm long. Muscles and periosteum covering the cranial bone were elevated to expose the underlying calvaria. A circular 5 mm defect was drilled in the center of the calvaria with a trephine (external diameter 5 mm, Moria) rotating at low speed, under irrigation with sterile saline to prevent thermal tissue damage. The endocranial surface was reached, taking care not to injure the underlying dura mater and midsagittal sinus. All 20 rats were randomly divided into four groups: sham group (operate and suture treatment, *n* = 5), blank group (cranial defect *n* = 5), PEG-DTT group (cranial defect + hydrogel treatment, *n* = 5), and PEG-DTT + AM1241 group (cranial defect + drug-loaded hydrogel treatment, *n* = 5). The defects were filled with either PEG-DTT hydrogels or the drug-loaded PEG-DTT hydrogels or remained empty according to different groups. Six weeks later, the rats were dealt with euthanasia, and the calvarium were collected for examination.

### Micro-CT Scanning (Micro-Computed Tomography)

At 6 weeks after cranial defect treatment, the rats were euthanized, removing the surrounding tissues, completely taking out the rat cranial bones. The cranial bone samples were immersed in 4% paraformaldehyde for fixation for 24 h. The micro-CT system was used to perform coronal, sagittal, and three-dimensional reconstruction scans to evaluate the bone healing of the cranial bone defect. Scanning equipment parameters: voltage 80 k Vp; current 500 mA. The size of the scanning observation area is 5 mm in diameter and 1.5 mm in thickness.

The relative bone mass (bone volume (BV)/tissue volume (TV), BV/TV), bone surface (BS) area to bone volume ratio (BS/TV), and trabecular bone number (Tb.N) were calculated by CTAn (Bruker MicroCT, Kontich, Belgium). CTVox software (Bruker MicroCT, Kontich, Belgium) was used to reconstruct the three-dimensional images.

### Histological Observation

The rat samples of cranial bone defects were collected and fixed with 4% paraformaldehyde solution for 24 h. Calvarium were decalcified using 14% ethylene diamine tetraacetic acid at pH 7.4 for 4 days. Then, the cranial bone was embedded in paraffin solution and cut longitudinally into a thickness of 5 μM. After the sections were made, performed them with H&E staining and Masson staining. Samples were also obtained for immunohistochemical (IHC) staining to analyze the expression of the vascular endothelial growth factor (VEGF), collagen type-1 (COL-1), and osteocalcin (OCN). Briefly, sodium citrate buffer was used to retrieve antigen for 20 min at 95°C, thus suppressing nonspecific binding for 1 h at room temperature. Samples were incubated with corresponding primary antibody overnight at 4°C and then incubated samples and secondary antibodies for 30 min. Samples were incubated with horseradish peroxidase-conjugated streptavidin (Vector Laboratories) and treated with the Vector NovaRED peroxidase substrate (Vector Laboratories). After IHC, samples were then subsequently counterstained with hematoxylin, dehydrated, mounted, and cover slipped. The images were captured by the Olympus CKX41 microscope.

### Statistical Analysis

All data were presented as average ± standard deviation. The results of the experiments were statistically analyzed using SPSS version 19.0 software. Statistical significance between two groups was evaluated by the two-tailed Student’s t test. The means of multiple groups were compared using one-way analysis of variance. *P* < 0.05 was considered significant.

## Results

### Morphology of Poly(ethylene glycol)–Dithiothreitol Hydrogels and its Thermosensitive Properties

**FIGURE 1:**
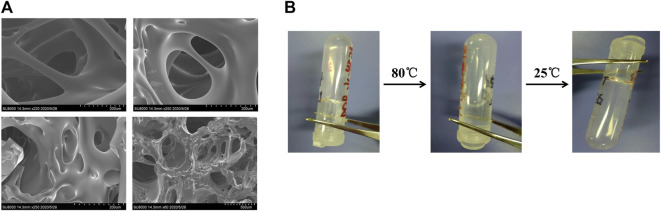
Preparation of hydrogels. **(A)** SEM images of PEGDA (molecular weight: 700) + DTT hydrogel catalyzed by 0.07 M borax. Sample was prepared by lyophilization. **(B)** Hydrogel formed from PEGDA (0.14 mM/ml) and DTT (0.14 mM/ml). Solution was produced after heating to 80°C. Hydrogel reformed after cooling the solution at 25°C.

As shown in Figure 1A, the dried PEG-DTT hydrogel had a porous morphology, which was a typical polymer hydrogel structure. The size of pore structure varied. Meanwhile, we could see the wall was smooth and thick. Thermosensitive hydrogels usually have a volume phase transition temperature (VPTT). When the temperature is higher than VPTT, the molecular segments in the gel become hydrophobic and be in liquid state. When the PEG-DTT hydrogel solution was heated to 80°C, the hydrogel showed in a liquid state. Subsequently, as the temperature dropped to 25°C, the state of the hydrogel gradually changed from the liquid to the gel state (Figure 1B).

### AM1241 Stimulated Osteoblast Differentiation *In Vitro*

**FIGURE 2:**
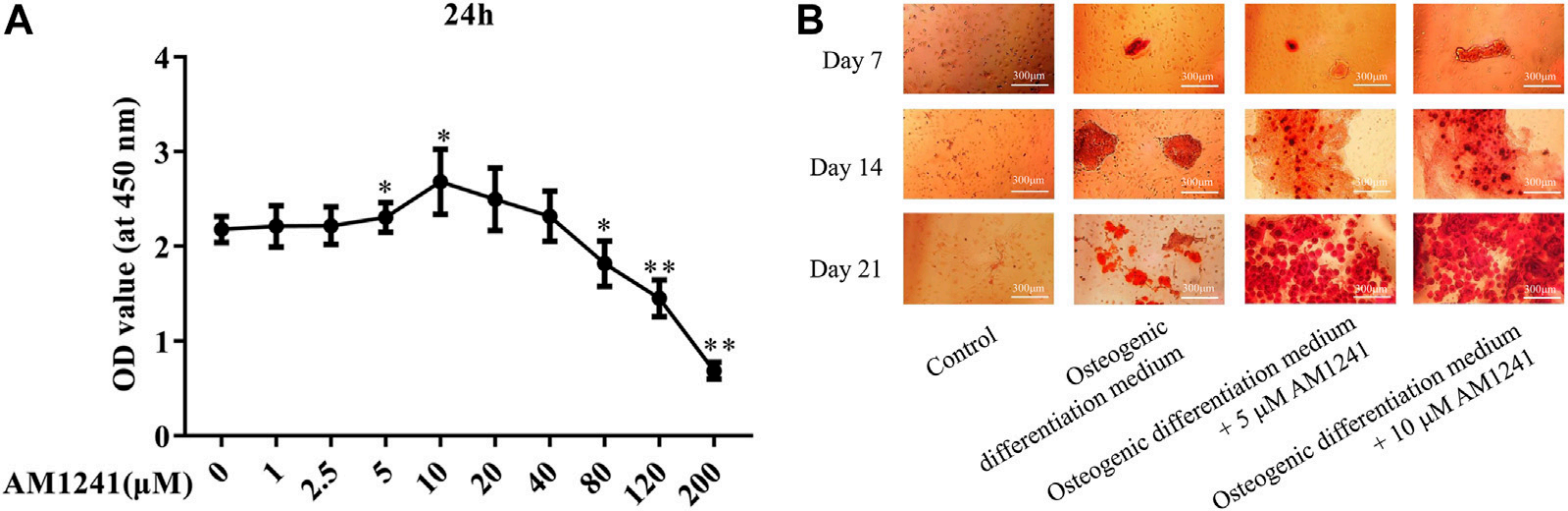
Effect of AM1241 on osteoblasts in vitro. **(A)** CCK-8 assay of determining the optimal concentration of AM1241 by the effect of AM1241 on the activity of BMSCs. **(B)** AR staining of osteogenic differentiation experiment of BMSCs.

We used the CCK-8 assay to assess the effect of AM1241 on the proliferative capacity of osteoblasts. The results showed that the cells exhibited the strongest viability when 5 μM or 10 μM AM1241 was added to BMSCs cells (*p* < 0.05, Figure 2A). Based on the best concentration experiment of the drug AM1241, we added 5 µM or 10 µM AM1241 to the osteogenic differentiation medium of BMSCs. The results of AR staining showed that the number of plaques of calcified osteoblasts increased with the increase of AM1241 concentration in BMSCs (Figure 2B). These results showed that a certain amount of AM1241 promoted osteoblast differentiation.

### AM1241-Loaded Poly(ethylene glycol)–Dithiothreitol Hydrogel Promotes Cranial Bone Injury in Rats

**FIGURE 3:**
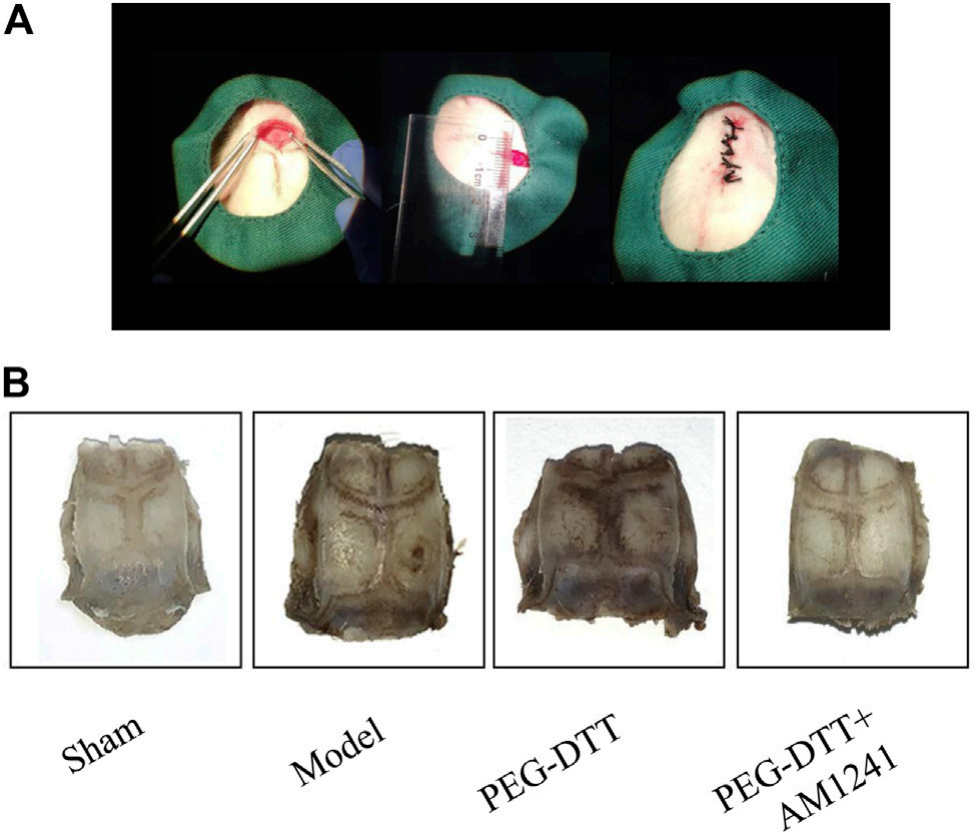
Construction of the rat cranial bone defect model. **(A)** Surgical steps of the rat cranial bone defect model. **(B)** General observation of the healing of cerebral bone defects.

We established a rat cranial bone injury model (Figure 3A). Meanwhile, we observed the healing of cranial defects (Figure 3B). Compared with the model group, the cranial defects in both the PEG-DTT group and the PEG-DTT + AM1241 group healed well.

**FIGURE 4:**
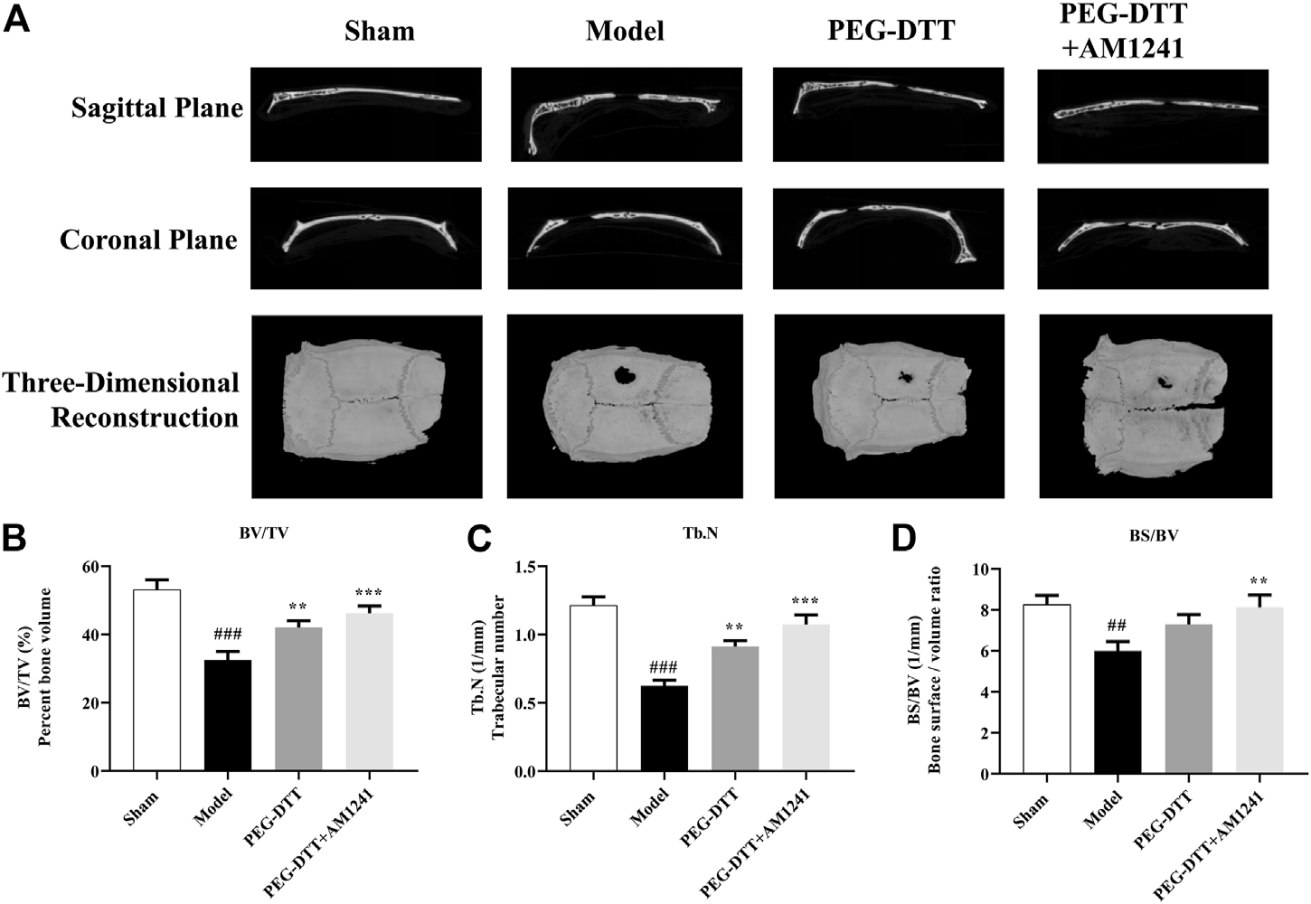
Micro-CT images and bone-related indexes. **(A)** Sagittal images, coronal images, and three-dimensional reconstruction of micro-CT scans from different groups. **(B)** Bone volume per total volume (BV/TV, %). **(C)** Trabecular number (Tb.N, 1/mm). **(D)** Bone surface per volume ratio (BS/BV). Compared with the Sham group, ^##^ *p* < 0.01, ^###^ *p* < 0.001; compared with the model group, **p < 0.01, ***p < 0.001.

After removing the implant and surrounding tissues, the sample was scanned with micro-CT. As shown in Figure 4A, the best defect recovery was observed in the PEG-DTT + AM1241 group compared to the model group. Similar results can also be clearly seen in the 3D images reconstructed from both the sagittal and coronal planes. Meanwhile, we calculated the bone-related indexes (Figures 4B–D). BV/TV, BS/TV, and Tb.N were significantly reduced in the model group compared with the blank group. In contrast, BV/TV and Tb.N were significantly higher in the PEG-DTT group compared with the model group. But there was no significant difference in BS/TV. Notably, the values of BV/TV, BS/TV, and Tb.N were significantly higher in the PEG-DTT + AM1241 group compared with the model group, indicating that the AM1241-loaded PEG-DTT hydrogel was more effective in repairing skull defects than when it was not loaded with the drug.

### Osteogenesis of Poly(ethylene glycol)–Dithiothreitol Hydrogel Loaded With AM1241

**FIGURE 5:**
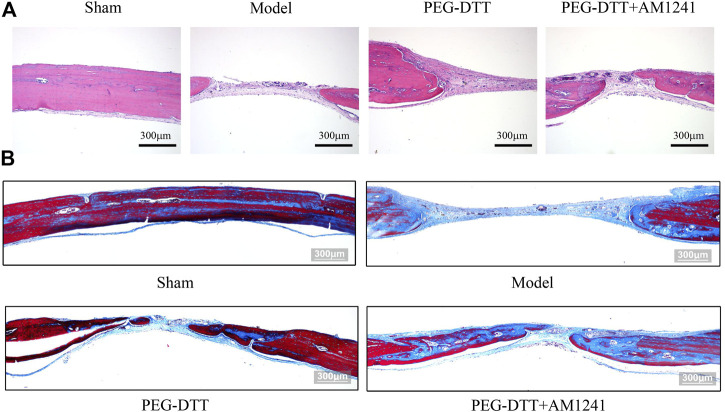
Histological staining images. **(A)** H&E staining. **(B)** Masson staining.

**FIGURE 6:**
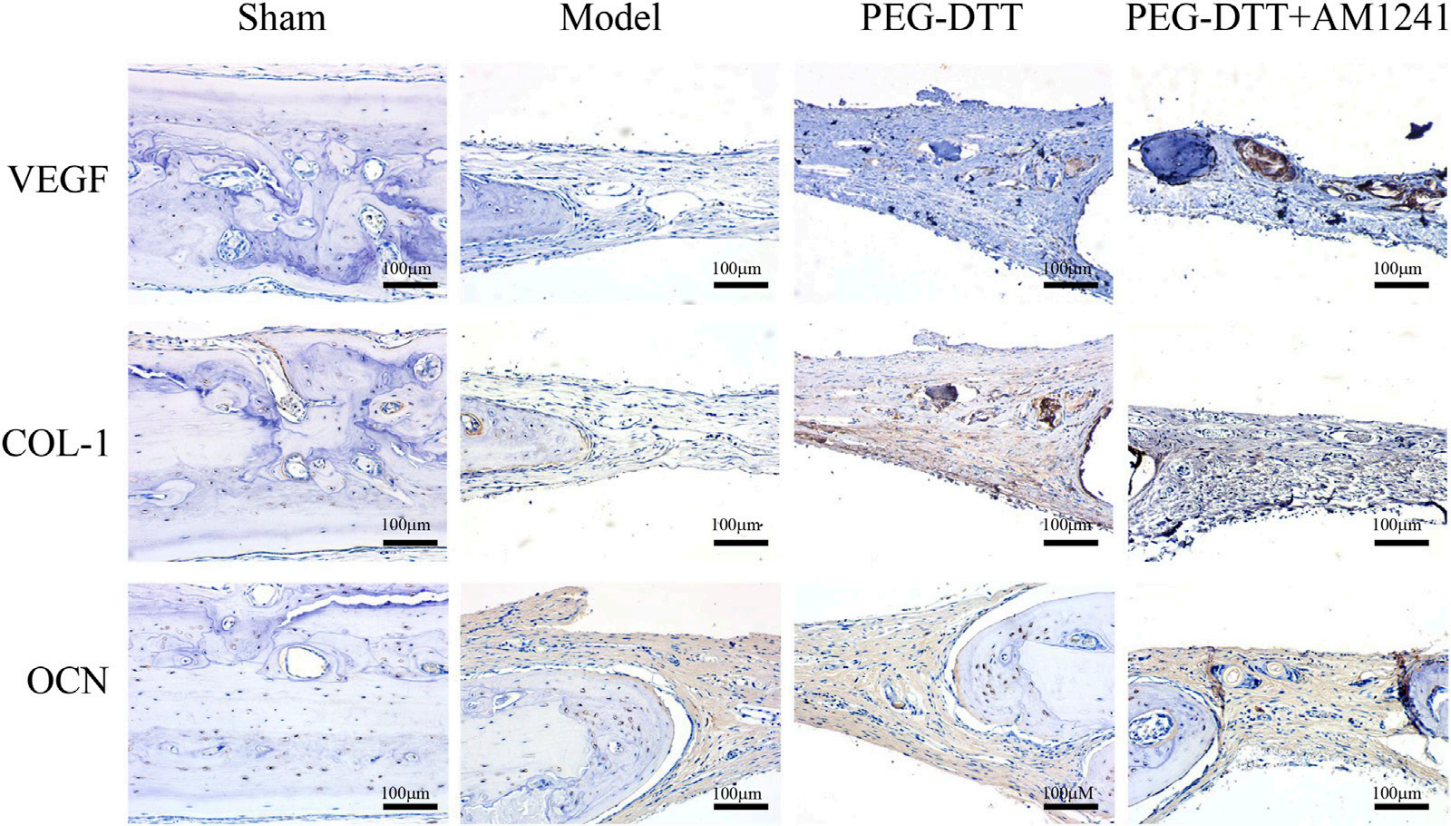
Immunohistochemistry staining of VEGF, COL-1 and OCN among the four groups, scale bar = 100 μm.

To clearly observe the effect of AM1241-loaded PEG-DTT hydrogel in inducing mineralization during osteogenesis, we performed histological staining. H&E staining showed that it occurred as a better repair of cranial fibrous connective tissue in the PEG-DTT and PEG-DTT + AM1241 groups of rats compared with the model group (Figure 5A). The results of Masson staining showed that there was new bone formation in the skull of rats in the PEG-DTT group and PEG-DTT + AM1241 group compared with the model group (Figure 5B). The results of both stainings showed that the AM1241-loaded PEG-DTT hydrogel had better effects on promoting fibrous tissue repair and new bone formation after skull injury compared with the PEG-DTT group. In addition, we also performed IHC staining. As shown in Figure 6, the protein expression of VEGF, COL-1, and OCN was significantly increased in both the PEG-DDT and PEG-DDT + AM1241 groups compared with the model group. The PEG-DDT + AM1241 group was higher than the others. Overall, the AM1241-loaded PEG-DDT hydrogel had excellent osteogenic effects on rats with cranial bone injury.

## Discussion

Bone tissue maintains its internal dynamic balance by continuously participating in the metabolic activities of the human body. When the balance between osteoblasts and osteoclasts is destroyed, it indicates that the body is in a state of disease or bone tissue cannot heal itself. To tackle the challenges of bone regeneration in clinical practice, a plenty of studies focus on seeking suitable biomaterials for implantation, so that bone cells can continually develop to repair bone defects (Wu et al., 2020; Zhang et al., 2020; Zhao et al., 2021; Dai et al., 2022). In our study, PEG-DTT hydrogel and PEG-DTT hydrogel loaded with AM1241 were prepared and established a rat cranial defect model to validate the effect of the hydrogel.

To date, many investigators are throwing themselves into the study of hydrogel development. For hydrogel, its soft texture can reduce the inflammatory response of surrounding cells and tissues, matching many soft biological tissues (Buwalda et al., 2017). According to the reaction of hydrogels to external stimuli, they can be classified as physical response hydrogels, chemical response hydrogels, and biochemical response hydrogels. Temperature-responsive hydrogels belong to physical response hydrogels, which can be rapidly gelled at physiological temperature at specific local tissues in BTE. The hydrogel prepared in our study belongs to thermosensitive hydrogels, which was liquid when heated to 80°C and gelatinous when cooled to 25°C, showing the characteristics of physical response hydrogels. Studies have shown that thermosensitive hydrogels have tumor-specific targeting, penetration, and release effects that can effectively prevent tumor recurrence (Liu et al., 2019). Tan et al. (2021) designed Cur-MP/IR820 gel to promote drug release and accelerate bone reconstruction in the treatment of bone tumors and repair of bone tissue. In the current study, the prepared PEG-DTT hydrogels could reach the defects sides and equally treat any shape of deformity (Parlato et al., 2014).

In addition, our assay results showed that PEG-DTT hydrogel could promote skull injury repair so as to achieve a better repair effect. Similarly, Yuan et al. designed the multiarm PEG-HA hydrogels, and it facilitated healing of the cranial bone defects more effectively in a Sprague-Dawley rat model after 10 weeks of implantation (Yuan et al., 2019). Hermann et al. (Hermann et al., 2014) reported on PEG hydrogels for the delivery of bone morphogenetic protein inhibitors to treat excessive postoperative bone regrowth (resynostosis) after surgical correction of premature fusion of cranial sutures (craniosynostosis) in children. Likewise, Nguyen et al. (2018) have pointed out that from *in situ* forming PEG hydrogels had the impact on bone formation in a rat calvarial defect model. Unusually, we have added AM1241 to PEG-DTT hydrogel. Cannabinoids maintained the balanced aspect between bone formation and bone resorption (Apostu et al., 2019). Thermal nociceptive hyperalgesia and mechanical hypersensitivity in bone cancer can be eliminated by using the CB-2 agonist AM1241 (Curto-Reyes et al., 2010). Besides, Kim et al. (2006) have pointed out that AM1241 had showed an effective effect in models of inflammation and hyperalgesia. In our study, the prepared hydrogel combined with AM1241 was used to treat cranial bone defects in rats. The results showed that the combination of hydrogel and AM1241 exerted a repairing effect on cranial bone injury by increasing the production of collagen fibers and the proliferation of osteoblasts compared with the PEG-DTT group. This is similar to previous studies that cannabinoids in primary human osteoblasts could act synergistically with drugs to promote their proliferative activity (Hojnik et al., 2015). It is supposed that AM1241-loaded PEG-DTT hydrogel promoted the expression of VEGF, COL-1, and OCN.

Studies have verified that some materials could effectively repair injured cranial bone tissues of calvarial defect rats through triggering osteogenic factors and its protein expression (Li et al., 2020; Zhou et al., 2020). Based on our findings, the protein expression of VEGF and COL-1 increased significantly in both PEG-DTT group and PEG-DTT + AM1241 group compared with the model group. As a key angiogenic growth factor, VEGF has been widely used in bone tissue engineering (Collin-Osdoby, 1994). VEGF binds its receptor on endothelial cells to stimulate blood vessel growth and bone regeneration. It also proved that the prepared hydrogel can promote osteogenesis. Wu et al. (2018) found that the expression of COL-1, osteopontin (OPN), and OCN proteins were enhanced in the combined drug group. However, our results showed that only PEG-DTT hydrogels loaded with AM1241 promoted the expression of OCN. Consistently, the study showed that COL-1 and OCN were determined significantly higher in drugs received groups (Lektemur Alpan et al., 2020). We considered that AM1241-loaded PEG-DTT hydrogel promoted osteoblast differentiation and enhanced vasculogenic differentiation.

## Conclusion

The prepared AM1241-loaded PEG-DTT hydrogel had a repairing effect on rat cranial defects by promoting the expression of osteogenic factors VEGF, COL-1, and OCN. These results provide a theoretical and practical basis for its further clinical application.

## Data Availability

The raw data supporting the conclusion of this article will be made available by the corresponding authors, per request.

## References

[B1] Apostu D. Lucaciu O. Mester A. Benea H. Oltean-Dan D. Onisor F. (2019). Cannabinoids and Bone Regeneration. Drug Metab. Rev. 51 (1), 65–75. 10.1080/03602532.2019.1574303 30702341

[B2] Buwalda S. J. Vermonden T. Hennink W. E. (2017). Hydrogels for Therapeutic Delivery: Current Developments and Future Directions. Biomacromolecules 18 (2), 316–330. 10.1021/acs.biomac.6b01604 28027640

[B3] Collin-Osdoby P. (1994). Role of Vascular Endothelial Cells in Bone Biology. J. Cel. Biochem. 55 (3), 304–309. 10.1002/jcb.240550306 7962161

[B4] Curto-Reyes V. Llames S. Hidalgo A. Menéndez L. Baamonde A. (2010). Spinal and Peripheral Analgesic Effects of the CB2cannabinoid Receptor Agonist AM1241 in Two Models of Bone Cancer-Induced Pain. Br. J. Pharmacol. 160 (3), 561–573. 10.1111/j.1476-5381.2009.00629.x 20233215 PMC2931557

[B16] Dai H. Hosseinpour S. Hua S. Xu C. (2022). Advances in Porous Inorganic Nanomaterials for Bone Regeneration. Nano. TransMed. 1 (1), e9130005. 10.26599/NTM.2022.9130005

[B5] Fenbo M. Sijing L. Ruiz-Ortega L. I. Yuanjun Z. Lei X. Kui W. (2020). Effects of Alginate/chondroitin Sulfate-Based Hydrogels on Bone Defects Healing. Mater. Sci. Eng. C 116, 111217. 10.1016/j.msec.2020.111217 32806290

[B6] Forrestal D. P. Klein T. J. Woodruff M. A. (2017). Challenges in Engineering Large Customized Bone Constructs. Biotechnol. Bioeng. 114 (6), 1129–1139. 10.1002/bit.26222 27858993

[B7] Galve-Roperh I. Chiurchiù V. Díaz-Alonso J. Bari M. Guzmán M. Maccarrone M. (2013). Cannabinoid Receptor Signaling in Progenitor/stem Cell Proliferation and Differentiation. Prog. Lipid Res. 52 (4), 633–650. 10.1016/j.plipres.2013.05.004 24076098

[B8] Hermann C. D. Wilson D. S. Lawrence K. A. Ning X. Olivares-Navarrete R. Williams J. K. (2014). Rapidly Polymerizing Injectable Click Hydrogel Therapy to Delay Bone Growth in a Murine Re-synostosis Model. Biomaterials 35 (36), 9698–9708. 10.1016/j.biomaterials.2014.07.065 25176067 PMC4217121

[B9] Hojnik M. Dobovišek L. Knez Ž. Ferk P. (2015). A Synergistic Interaction of 17-β-Estradiol with Specific Cannabinoid Receptor Type 2 Antagonist/inverse Agonist on Proliferation Activity in Primary Human Osteoblasts. Biomed. Rep. 3 (4), 554–558. 10.3892/br.2015.469 26171165 PMC4487012

[B10] Hotta R. Cheng L. S. Graham H. K. Nagy N. Belkind-Gerson J. Mattheolabakis G. (2016). Delivery of Enteric Neural Progenitors with 5-HT4 Agonist-Loaded Nanoparticles and Thermosensitive Hydrogel Enhances Cell Proliferation and Differentiation Following Transplantation *In Vivo* . Biomaterials 88, 1–11. 10.1016/j.biomaterials.2016.02.016 26922325 PMC4792702

[B11] Kim K. Moore D. H. Makriyannis A. Abood M. E. (2006). AM1241, a Cannabinoid CB2 Receptor Selective Compound, Delays Disease Progression in a Mouse Model of Amyotrophic Lateral Sclerosis. Eur. J. Pharmacol. 542 (1-3), 100–105. 10.1016/j.ejphar.2006.05.025 16781706

[B12] Kim Y.-H. Furuya H. Tabata Y. (2014). Enhancement of Bone Regeneration by Dual Release of a Macrophage Recruitment Agent and Platelet-Rich Plasma from Gelatin Hydrogels. Biomaterials 35 (1), 214–224. 10.1016/j.biomaterials.2013.09.103 24125774

[B13] Lavanya K. Chandran S. V. Balagangadharan K. Selvamurugan N. (2020). Temperature- and pH-Responsive Chitosan-Based Injectable Hydrogels for Bone Tissue Engineering. Mater. Sci. Eng. C 111, 110862. 10.1016/j.msec.2020.110862 32279825

[B14] Lektemur Alpan A. Çalişir M. Kizildağ A. Özdede M. Özmen Ö. (2020). Effects of a Glycogen Synthase Kinase 3 Inhibitor Tideglusib on Bone Regeneration with Calvarial Defects. J. Craniofac. Surg. 31 (5), 1477–1482. 10.1097/scs.0000000000006326 32195836

[B15] Li J. Liao G. Long Z. Qiu P. Ding L. Xiong L. (2020). Study of PLGA Microspheres Loaded with pOsx/PEI Nanoparticles for Repairing Bone Defects *In Vivo* and *In Vitro* . Adv. Clin. Exp. Med. 29 (4), 431–440. 10.17219/acem/116752 32364686

[B17] Liang T. Wu J. Li F. Huang Z. Pi Y. Miao G. (2020). Drug‐loading Three‐dimensional Scaffolds Based on Hydroxyapatite‐sodium Alginate for Bone Regeneration. J. Biomed. Mater. Res. 109, 219–231. 10.1002/jbm.a.37018 32490561

[B18] Liu H. Shi X. Wu D. Kahsay Khshen F. Deng L. Dong A. (2019). Injectable, Biodegradable, Thermosensitive Nanoparticles-Aggregated Hydrogel with Tumor-specific Targeting, Penetration, and Release for Efficient Postsurgical Prevention of Tumor Recurrence. ACS Appl. Mater. Inter. 11 (22), 19700–19711. 10.1021/acsami.9b01987 31070356

[B19] Lu X. Perera T. H. Aria A. B. Smith Callahan L. A. (2018). Polyethylene Glycol in Spinal Cord Injury Repair: a Critical Review. Jep 10, 37–49. 10.2147/jep.s148944 PMC6067622 30100766

[B20] Nguyen M. K. Jeon O. Dang P. N. Huynh C. T. Varghai D. Riazi H. (2018). RNA Interfering Molecule Delivery from *In Situ* Forming Biodegradable Hydrogels for Enhancement of Bone Formation in Rat Calvarial Bone Defects. Acta Biomater. 75, 105–114. 10.1016/j.actbio.2018.06.007 29885529 PMC6119505

[B21] Parlato M. Reichert S. Barney N. Murphy W. L. (2014). Poly(ethylene Glycol) Hydrogels with Adaptable Mechanical and Degradation Properties for Use in Biomedical Applications. Macromol. Biosci. 14 (5), 687–698. 10.1002/mabi.201300418 24949497 PMC4066198

[B22] Tan B. Wu Y. Wu Y. Shi K. Han R. Li Y. (2021). Curcumin-Microsphere/IR820 Hybrid Bifunctional Hydrogels for *In Situ* Osteosarcoma Chemo-Co-Thermal Therapy and Bone Reconstruction. ACS Appl. Mater. Inter. 13 (27), 31542–31553. 10.1021/acsami.1c08775 34191477

[B23] Wu G. Feng C. Quan J. Wang Z. Wei W. Zang S. (2018). *In Situ* controlled Release of Stromal Cell-Derived Factor-1α and antimiR-138 for On-Demand Cranial Bone Regeneration. Carbohydr. Polym. 182, 215–224. 10.1016/j.carbpol.2017.10.090 29279118

[B24] Wu X. Zhang T. Hoff B. Suvarnapathaki S. Lantigua D. McCarthy C. (2020). Mineralized Hydrogels Induce Bone Regeneration in Critical Size Cranial Defects. Adv. Healthc. Mater. 10, 2001101. 10.1002/adhm.202001101 32940013

[B25] Yuan J. Maturavongsadit P. Metavarayuth K. Luckanagul J. A. Wang Q. (2019). Enhanced Bone Defect Repair by Polymeric Substitute Fillers of MultiArm Polyethylene Glycol‐Crosslinked Hyaluronic Acid Hydrogels. Macromol. Biosci. 19 (6), 1900021. 10.1002/mabi.201900021 30942959

[B26] Zhang J. Shi H. Zhang N. Hu L. Jing W. Pan J. (2020). Interleukin‐4‐loaded Hydrogel Scaffold Regulates Macrophages Polarization to Promote Bone Mesenchymal Stem Cells Osteogenic Differentiation via TGF‐β1/Smad Pathway for Repair of Bone Defect. Cell Prolif. 53, e12907. 10.1111/cpr.12907 32951298 PMC7574882

[B27] Zhao D. Zhu T. Li J. Cui L. Zhang Z. Zhuang X. (2021). Poly(lactic-co-glycolic Acid)-Based Composite Bone-Substitute Materials. Bioactive Mater. 6 (2), 346–360. 10.1016/j.bioactmat.2020.08.016 PMC7475521 32954053

[B28] Zhou C. Ye C. Zhao C. Liao J. Li Y. Chen H. (2020). A Composite Tissue Engineered Bone Material Consisting of Bone Mesenchymal Stem Cells, Bone Morphogenetic Protein 9 (BMP9) Gene Lentiviral Vector, and P3HB4HB Thermogel (BMSCs-LV-BMP9-P3hb4hb) Repairs Calvarial Skull Defects in Rats by Expression of Osteogenic Factors. Med. Sci. Monit. 26, e924666. 10.12659/msm.924666 32894745 PMC7496453

